# REAC neurobiological treatments in acute post-traumatic knee medial collateral ligament lesion

**DOI:** 10.1016/j.heliyon.2020.e04539

**Published:** 2020-07-24

**Authors:** Ana Rita Pinheiro Barcessat, Marina Nolli Bittencourt, Jose Alfredo Coelho Pereira, Alessandro Castagna, Vania Fontani, Salvatore Rinaldi

**Affiliations:** a Programa de Pós Graduação em Ciências da Saúde, Universidade Federal do Amapá, Macapá, Brazil; b Research Department, Rinaldi Fontani Foundation, Florence, Italy; c Department of Neuro Psycho Physio Pathology and Neuro Psycho Physical Optimization, Rinaldi Fontani Institute, Florence, Italy; d Department of Regenerative Medicine, Rinaldi Fontani Institute, Florence, Italy

**Keywords:** Health sciences, Musculoskeletal system, Behavioral medicine, Evidence-based medicine, Clinical research, Knee, Medial collateral ligament, Functional recovery, Psychomotor performance

## Abstract

**Objective:**

Physical traumas can lead to unconscious neuropsychical alterations, which can compromise rehabilitation result and functional recovery. Aim of this interventional study is to verify if neurobiological Radio Electric Asymmetric Conveyer (REAC) treatments Neuro Postural Optimization (NPO) and Tissue Optimization (TO) are able respectively to improve neuro psychomotor strategies and facilitate recovery process in medial collateral ligaments (MCL) lesions of the knee.

**Patients and methods:**

45 healthy subjects, 32 males and 13 females, with knee MCL lesion, diagnosed with MRI or ultrasound. Within 4 days after the trauma, subjects were clinically evaluated (T0), both through medical and subjective assessments. Clinical evaluation was repeated after the REAC NPO treatment (T1) and at the end of 18 REAC TO treatments (T2) and at the 30 days follow-up (T3).

**Results:**

In comparison with the results commonly found in clinical practice, all REAC treated patients recovered much faster. They reported functional recovery, pain relief and joint stability, regardless of the severity of the lesion.

**Conclusion:**

The combined use of REAC NPO and TO can envisage a new rehabilitative approach, which aims not only at recovering the outcomes of the physical trauma, but also at improving the neuropsychical state that can condition the rehabilitation result.

## 1 Introduction

The most common injuries are those of the medial collateral ligament (MCL) [1, 2, 3], produced by distortion of the knee with valgus deviation and external rotation. This injury is particularly frequent especially in professional athletes [4, 5]. The lesion of the ligament may be partial (grade 2 MCL lesion) or complete (grade 3 lesion). As reported in literature, partial recovery time is around 4–5 weeks for grade 2 MCL lesion and at least 8 weeks for grade 3 MCL lesion. Usually, the treatment for these lesions is conservative. The knee joint should be immobilized in 25-degree flexion for about 10–25 days, depending on the severity of the lesion. Rehabilitation treatment sometimes requires several months [6]. Every type of joint lesion generates both a physical and neuropsychical response. The neuropsychical response often tends to also affect the success of a proper physical rehabilitation [7, 8, 9]. Therefore, it would be useful a rehabilitative approach which aims at recovering not only the outcomes of the physical trauma, such as injured tissues, but also the neuropsychical state that can condition the complete recovery [7, 10]. Recent studies show that people suffering joint lesions tend to experience more depression [11, 12], anxiety [13], emotional impairment [14], have a fear of re-injury [15, 16, 17], have decreased sport satisfaction [17]. This would suggest that joint lesions produce not only a physical but also a neuropsychical impact on injured people. The REAC technology platform carries out therapeutic protocols of neuro and bio modulation to optimize the neuro biological response. In the field of neuromodulation, REAC technology deliveries various treatment protocols, including the Neuro Postural Optimization (NPO), which showed to be able to produce long lasting effects in reshaping the average patterns of brain activation. Functional MRI performed during motor tasks showed that the brain areas involved in the task were reduced both in extension and degree of activation [18, 19, 20]. Moreover, REAC NPO showed to have long lasting effects in inducing neuromotor coordination of the lower limbs, through the disappearance of functional dysmetria (FD) [18, 19, 20], improving fine motor control [21] and balance [22], in healthy peoples and also in neurodegenerative diseases like Alzheimer [23]. NPO has also been shown to have effects in improving depression, anxiety and stress [24].

In the field of biomodulation, specific tissue optimization (TO) treatment protocols with REAC technology are effective not only in post-traumatic outcomes [25, 26], regenerative medicine [27, 28, 29], and in neurodegenerative diseases [30, 31, 32, 33], but also in direct cellular reprogramming [34, 35, 36]. TO treatments have been used for years, both in human and veterinary medicine, in the treatment of post-traumatic lesions in various types of tissues, as muscle, ligament and tendon; nevertheless, scientific work on this topic had never been produced to date. On these premises, in this study we have used REAC technology NPO and TO treatments in post-traumatic injuries of knee ligaments and we have chosen to treat in particular MCL lesions as these are among the most common joint lesions.

Aim of this study is to verify if REAC neurobiological treatments are able to facilitate recovery process in MCL lesions of the knee. In particular, the hypothesis is that NPO and TO treatments are able respectively to improve neuro psychomotor strategies and the neuro psycho physical response to trauma.

## 2 Materials and methods

### 2.1 Ethics

This study was approved by the Ethics Committee of the Federal University of Amapá, Brazil with the number 3.238.424. Clinical investigations and procedures mentioned in this paper are in accordance with the ethical standards and with the Helsinki Declaration and revisions. Patients have given their informed consent for participation in the research study.

### 2.2 Endpoints

The primary objective of this study was to assess the efficacy of REAC neurobiological treatments in facilitating the healing processes in MCL lesions of the knee. The Valgus Stress Test at 0° and 30° of flexion was selected as primary variable, since 100% of subjects were positive to this test at baseline T0 and this diagnostic tool allowed us to test the MCL as the primary stabilizer, and the joint capsule, the medial joint capsule, and anterior and posterior cruciate ligaments, evaluating the overall knee function.

The primary endpoint of this study was therefore to measure the proportion of patients who responded to REAC treatments improving in valgus stress test both at 0° and 30° and showing a complete recovery of the global function of the injured knee at the end of the trial.

As Additional Primary Endpoints, further medical assessments (Functional dysmetria test, Joint swelling assessment, Patellar ballottement assessment, Knee flexion angle) were performed in order to measure the percentage of patients responding to REAC treatments at each time point.

In order to evaluate the healing process also from the self-perceptive point of view, Subjective assessments of symptoms (Functional impotence, Pain, Feeling of stiffness, Feeling of knee instability, Monopodal instability) were conducted as Secondary Endpoints to measure the proportion of patients who responded to REAC treatments and presented an improvement in the evaluation scale of symptoms perception.

### 2.3 Patients

The participants were enrolled among the outpatients in care at Health Sciences Department of Federal University of Amapá, Macapá – Brazil, according to the following eligibility criteria:

Inclusion criteria: age 18–70 years; injury to the medial collateral ligament (MCL) of the knee for sports trauma and road or work accident; diagnosis of grade 3 or 2 lesion of the MCL of the knee joint, made by ultrasound or MRI scan no more than 4 days before the assessment for eligibility.

Exclusion criteria: history records of other ligamentous or capsular traumas or problems related to knee hyperextension.

50 patients met the eligibility criteria and were enrolled for the study. All these 50 patients were administered the entire intended treatments cycle and no discontinued intervention was recorded. Since 5 patients did not show at the 30-day follow-up, the results analysis was conducted on 45 subjects, 32 males and 13 females, with age ranging from 18 to 67 years, mean age of 35 ± 12.97.

Among the 45 patients, 23 (17 males and 6 females) had a diagnosis of grade 3 MCL lesion (20 from sport accident, 2 from work accident and 1 from road accident), while 22 (15 males and 7 females) had a diagnosis of grade 2 MCL lesion (14 from sport accident, 5 from work accident and 3 from road accident).

No one of the 45 subjects had history records of other ligamentous or capsular traumas or problems related to knee hyperextension. At the time of the first medical examination, all the patients reported impairments, including pain, swelling, loss of motion, weakness, joint laxity, and loss of proprioception. Functional limitations included limitations in performing basic activities of daily living and sports, such as difficulty with walking and climbing stairs and the inability to run and jump. Beyond the signs described above, referable to the trauma, all the subjects presented a disorder of the neuromotor control of the lower limbs, called functional dysmetria. Dysmetria, sometimes confused with heterometry, literally different measure, is an altered execution of voluntary movements, which can occur as a result of dysfunctions or lesions of the cerebellum and/or spinal cord [37]. It is opportune to remember that the dysmetria, in addition to involving the movement also involves thought and emotion [38] (emotional, affective, cognitive and relational functions). When the dysmetria is present in healthy subjects [20], it is defined functional [18] and is correlated to phenomena of adaptive type at the base of the fluctuating asymmetry [39].

After the first clinical examination and throughout the duration of the study, patients did not receive any type of treatment, neither pharmacological nor rehabilitative, except for REAC treatments. None of the patients had undergone reconstructive surgery.

#### 2.3.1 Patients clinical evaluation

The clinical evaluation of all the patients with both grades of lesion was done at baseline (T0, within 4 days after the trauma), immediately after the NPO administration (T1), at the end of 18 sessions of TO treatment (T2, within 2 weeks maximum from T0) and at 30 days follow-up (T3). The clinical evaluation of the patients was performed through 10 assessments: 5 medical assessments made by clinicians (Functional dysmetria test, Joint swelling assessment, Patellar ballottement assessment, Valgus Stress Test at 0° and 30°, Knee flexion angle) and 5 subjective assessments made by the patients themselves (Functional impotence, Pain, Feeling of stiffness, Feeling of knee instability, Monopodal instability test).

### 2.4 Medical assessments

#### 2.4.1 Functional dysmetria

The evaluation of functional dysmetria (FD) is an evaluation of neurological semiology aimed at observing the presence of a dysfunction. FD in the lower limbs can be evidenced by symmetrically placing the operator's hands on the femoral quadriceps of the subject being examined in supine position, taking care that the nails of the two left and right thumbs are perfectly aligned. Asking the subject to move from the supine to the sitting position, a progressive misalignment of the two thumbs is highlighted. This manoeuvre allows the operator to perceive and highlight the asymmetric activation of symmetrical muscle groups (dysmetria), such as the quadriceps muscles.

#### 2.4.2 Joint swelling assessment

The scale none (1), little (2), very (3), very much (4), was used to assess the symptom Joint swelling.

#### 2.4.3 Patellar ballottement assessment

The scale none (1), little (2), very (3), very much (4), was used to assess the patellar ballottement.

#### 2.4.4 Valgus stress test

The following scale was used to assess the increased motion: none (1) = level 1: 0–5 mm of joint opening, no instability; little (2) = level 2: 5–10 mm of joint opening, mild instability; very (3) = level 3: 10–15 mm of joint opening, moderate instability; very much (4) = level 4, >15 mm of joint opening, severe instability.

#### 2.4.5 Knee flexion angle

The knee flexion angle was measured in degrees with Ortho physical lite, a knee goniometer application [40], installed in an Apple iPhone© 6s.

#### 2.4.6 Subjective assessments of symptoms

The scale none (1), little (2), very (3), very much (4) was used for the evaluation of the following symptoms assessed subjectively by patients: Functional impotence, Pain, Feeling of stiffness, Feeling of knee instability, Monopodal instability.

### 2.5 REAC technology description

The REAC is a technology platform for biomodulation [35, 41] and neuromodulation [20, 24, 33] treatments. The REAC technology device used in this clinical investigation was a BENE 110 model (ASMED, Florence, Italy). The study was conducted administering 2 treatments with the REAC device: the Neuro Postural Optimization (NPO) and the Tissue Optimization (TO).

#### 2.5.1 REAC neuromodulation treatments

In this study, we used a REAC neuromodulation treatment: the Neuro Postural Optimization (NPO), that consists of a single treatment of 250 ms administered on a specific area of the auricle pavilion [18, 19, 20]. NPO is normally administered only once and it has been shown to stably induce a brain modulation, resulting in an improvement of motor strategies [18, 20, 21, 22], even in subjects with advanced Alzheimer's disease [23].

#### 2.5.2 REAC biomodulation treatments

The REAC biomodulation protocols take the name of Tissue Optimization (TO) [25, 26, 27, 28, 29, 31, 34, 35, 36, 41]. The primary aim of the TO treatments is to promote reparative and regenerative processes in various organs and tissues. Depending on the type, severity and depth of the lesion, TO treatments have different times and methods of administration. The TO treatments are administered by applying the device probe, which takes the name of Asymmetric Conveyer Probe (ACP), on the area to be treated. The ACP is held in place by a tubular elastic bandage. In this study, we administered 18 sessions of reparative TO treatment within a maximum of 2 weeks.

### 2.6 Statistical analysis

The clinical evaluation of patients is the primary objective of the study and the Valgus stress test was selected as primary endpoint. Considering positive patients for the Valgus stress test at 0° and 30° degrees of flexion, the assumption was to observe at least 90% of patients having an improvement in both degrees of flexion at time T2. The sample size of 45 patients was estimated to be able to calculate a 2-sided 95% confidence interval for the proportion of 90% with a precision of ±8.8%. Accounting for a drop-outs rate of 10%, the sample size was increased to 50 patients.

Individual medical assessments were described using descriptive statistics presenting number of patients and percentages at each time point. A subgroups descriptive analysis was presented for patients with grade 3 and grade 2 lesions.

The continuous variables of the symptoms were measured and approximated with Gaussian curves. The curves of the values of each symptom, at T0, T2, T3, were shown on the same graph so as to be able to visually evaluate the correlation. SPSS software, version 13.00, was used for statistical evaluations.

## 3 Results

### 3.1 Medical assessments

#### 3.1.1 Functional dysmetria

At baseline T0, functional dysmetria (FD) was present in 100% of the patients (45/45). The FD evaluation performed immediately after the administration of NPO treatment (T1) showed the disappearance of the symptom in 100% of the patients. At T2 and T3, the stable disappearance of the functional dysmetria was confirmed in 100% of the patients.

#### 3.1.2 Joint swelling assessment

Joint swelling was present only in 33 patients out of 45 (about 73% of all patients).

At T2, 55,6% of the total number of patients (25 out of 45) reported a reduction of the symptom.

At T3, 37,8% of them (17 out of 45) reported further reduction of the symptom.

These percentages need to be reconsidered taking into account that only 73% of patients had the symptom. In order to correctly evaluate the efficacy of the treatments, it is necessary to relate the results to the actual number of patients presenting the symptom Joint swelling at T0.

Consequently, considering only the 33 patients presenting the symptom, over 75% (25 out of 33) benefited from REAC treatment at T2, reporting a reduction of the joint swelling. At T3, about 48% of patients (16 out of 33 presenting the symptom) remain stable without further improvements.

Analysing the results by grade of lesion, all the 23 patients with a grade 3 MCL lesion presented joint swelling. In this group, at T2 REAC treatments have been shown to be effective in reducing joint swelling in about 74% of patients (17 out of 23). At the 30 days follow-up (T3), out of the total number of patients with the grade 3 MCL lesion, 39% (9 out of 23) remain stable, while 61% (14 out of 23) reported further reduction of the symptom.

As regards the group with the grade 2 MCL lesion, only 10 patients out of 22 reported joint swelling (about 45%). Out of a total of 10 patients presenting the symptom, at T2 REAC treatments have been shown to be effective in reducing joint swelling in 80% of patients (8 out of 10). At the 30 days follow-up (T3), 70% (7 patients out of 10 presenting the symptom) remain stable and 30% reported further reduction of the symptom.

**Figure 1:**
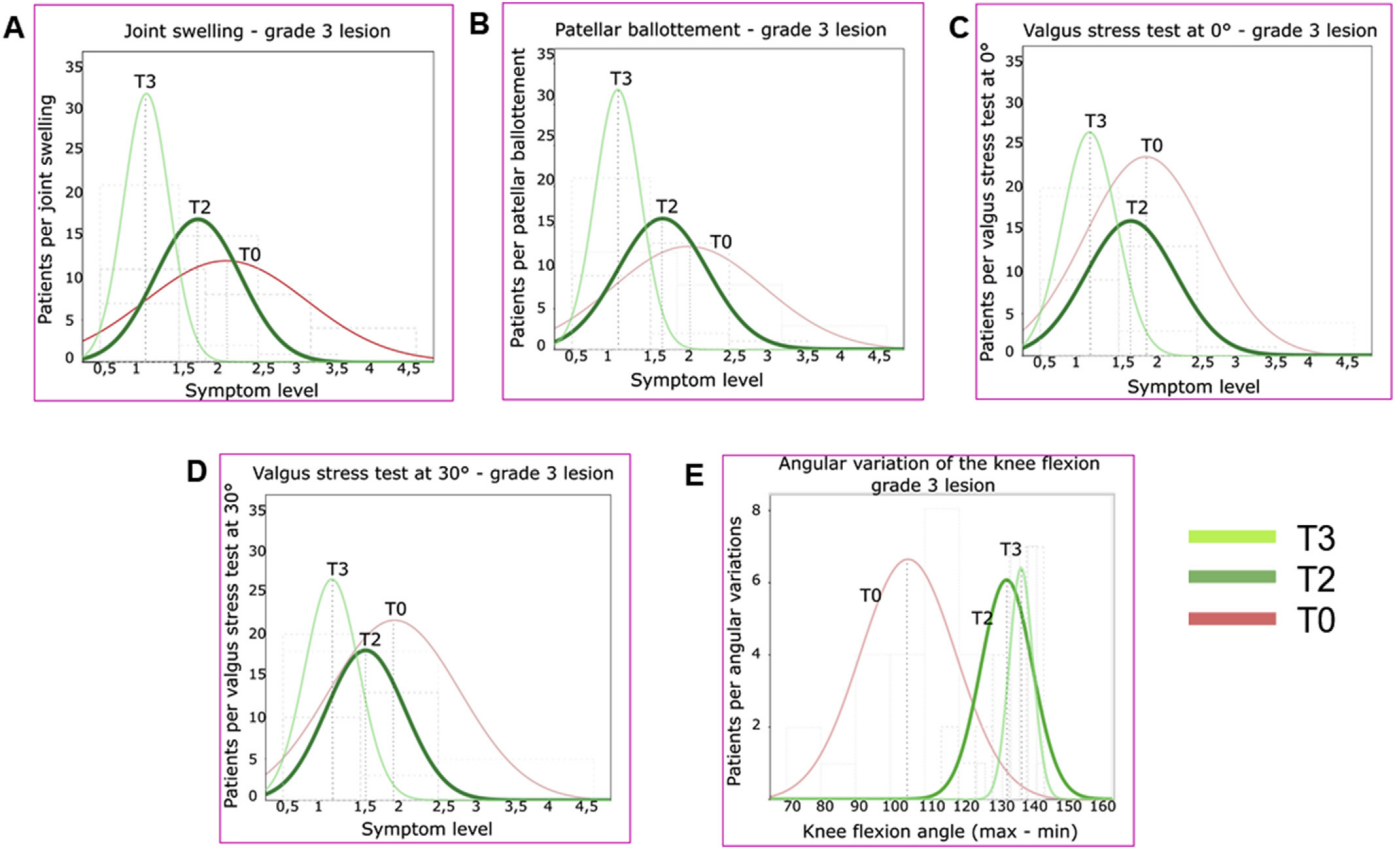
REAC efficacy in clinical assessments of symptoms represented by Gaussian distribution of grade 3 lesion patients from T0 to T3. REAC efficacy represented by the trend towards the distribution of patients on milder symptom levels.

**Figure 2:**
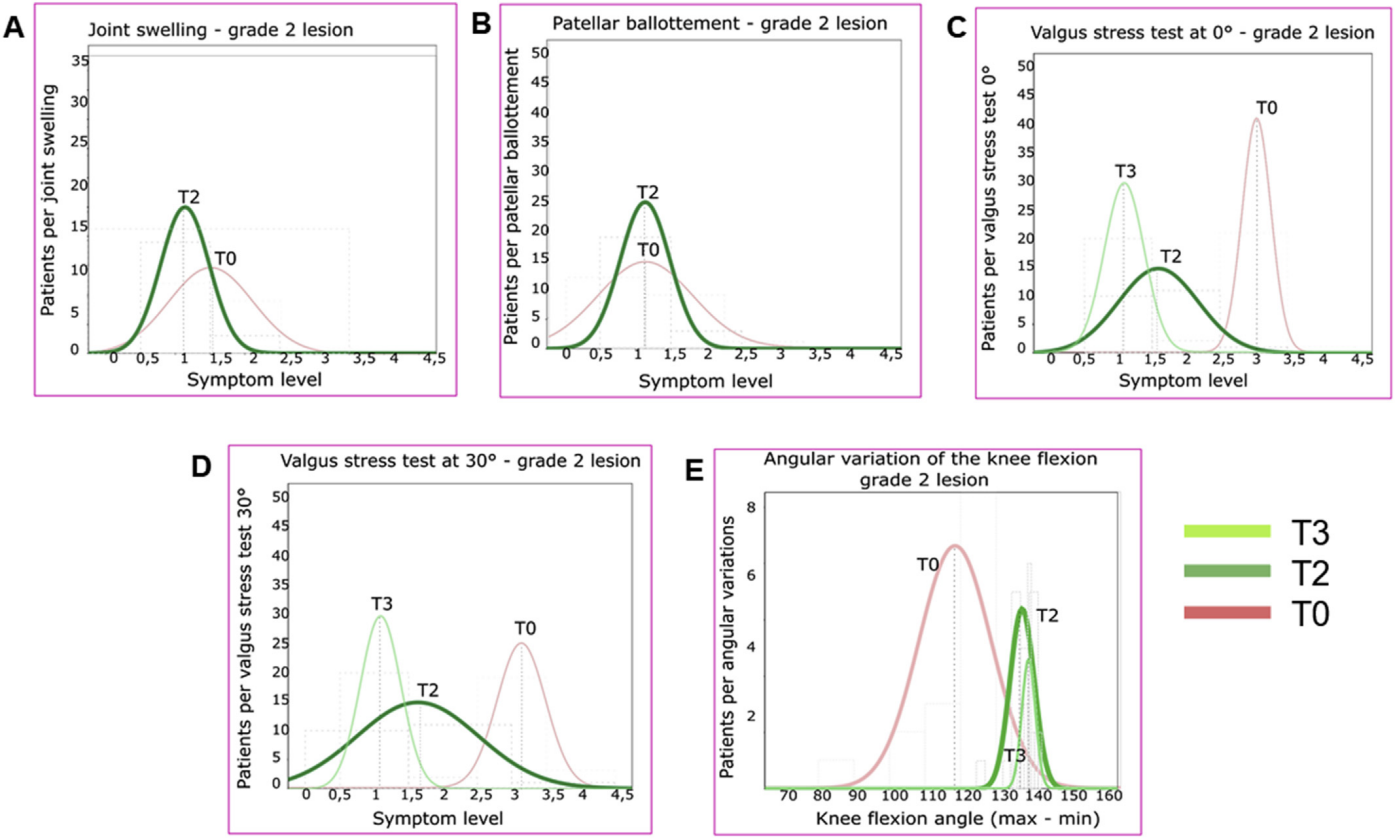
REAC efficacy in clinical assessments of symptoms represented by Gaussian distribution of grade 2 lesion patients from T0 to T3. REAC efficacy represented by the trend towards the distribution of patients on milder symptom levels.

The efficacy of the REAC treatment is confirmed for both grade of lesion by the trend represented in the graphs of Figures 1A and 2A, where between T0 and T2 the Gauss curve moves towards zero and assumes a less enlarged form, meaning that the patients (number in ordinate) after the REAC treatment tend to gather on milder symptom levels (abscissa values).

This trend towards the distribution of patients on milder symptom levels is confirmed by the T3 curves. In particular, as regards grade 2 MCL lesions, the T3 graph is not visible, since the totality of the patients showed full recovery and placed in correspondence of the absence of symptom in the values of the abscissa.

#### 3.1.3 Patellar ballottement assessment

The symptom Patellar ballottement is closely related to the presence of joint swelling, as local oedema raises the patella further off the femur, causing an increase in the anterior to posterior motion. This is why the results for this symptom are consistent with those observed in the clinical evaluation of the symptom joint swelling.

The efficacy of the REAC treatment is confirmed for both grades of lesion by the trend represented in the graphs of Figures 1B and 2B, which are consistent to those relating to the symptom Joint swelling.

#### 3.1.4 Valgus stress test - extended 0° and flexed 30° knee

At baseline (T0), all 45 patients were positive at Valgus Stress Test, both at 0° and at 30° of flexion.

At the end of 18 sessions of TO treatment (T2), REAC treatments have been shown to be effective in reducing the symptom in both degrees of flexion in about 95,6% of patients (43 out of 45), with 95%CI: 0,956 (0,896; 1,016).

At the 30 days follow-up (T3), 46,7% of patients (21 out of 45) reported further improvement both at 0° and at 30° of flexion, with 95%CI: 0,466 (0,320; 0,612).

Analysing the results by grade of lesion, in the evaluation at 0° degree, about 96% of patients with grade 3 MCL lesion (22 patients out of 23) reported at T2 a reduction of the symptom, while at T3 43% of patients remained stable and 57% reported further reduction of the symptom. As regards the results of the Valgus stress test at 30° degree in the same 3-grade-lesion-group, at T2 REAC treatments were effective in 100% of patients (23 out of 23), while at T3 57% of patients (13 out of 23) remain stable and 43% reported further reduction of the symptom.

In the group with grade 2 MCL lesion, at T2 the results of the Valgus stress test at 0° degree showed that REAC treatments were effective in about 95% of patients (21 out of 22), while at T3 50% of patients remain stable and 50% reported a reduction of the symptom. As regards the results of the Valgus stress test at 30° degree in the same group with grade 2 lesion, at T2 REAC treatments showed to be effective in about 95% of patients (21 out of 22), while at T3 50% of the patients remain stable and 50% reported further reduction of the symptom.

In the graphs of Figures 1C, D and 2C, D, for both 3 and 2 grade lesions and for both 0° and 30° Valgus Stress Test, the Gauss Curves show a remarkable shift towards milder symptom levels (abscissa values) immediately after REAC treatment (T2), albeit with a non-homogeneous distribution of patients, and a further consolidation of the results at T3.

#### 3.1.5 Knee flexion angle

At T0, the reduction of the knee flexion angle was present in all the 45 patients.

Considering 0°/140° as the standard range in healthy knees, at T2 REAC treatments showed to be effective in 100% of patients, improving the range of excursion of the knee towards nearly normal values, as described in Table 1.

**Table 1:** Knee angle test: results of knee angle test in grade 3 and 2 lesion patients at T0, T2 and T3.

Knee Flexion Angle Test			
Grade 3 Lesion		Grade 2 Lesion	
Average knee flexion angle		Average knee flexion angle	
**T0**		**T0**	
7,65°	112,82°	7,59°	126,04°
**T2**		**T2**	
1,52°	135,69°	1,36°	139,31°
**T3**		**T3**	
0,17°	138,95°	0,13°	139,90°

In the graphs of Figures 1E and 2E, for both 3 and 2 grade lesions, the Gauss Curves show a remarkable shift towards a wider range of knee flexion values (abscissa values) immediately after REAC treatment (T2). The recovery of patients is almost complete, as evidenced by the fact that at T3 the curve nearly overlaps the same values of T2.

### 3.2 Subjective assessments of symptoms

#### 3.2.1 Functional impotence

At T0, functional impotence was present in all the 45 patients.

At T2, self-assessment tests reported a reduction of the symptom in 100% of patients, while at T3 44% of patients reported further improvements.

More in detail, at T2 REAC treatments have been shown to be effective in 100% of patients with grade 3 MCL lesion, while at T3 57% of patients remain stable and 43% reported further reduction of the symptom. Similarly, at T2 the REAC treatments were effective in 100% of patients with grade 2 MCL lesion, while at T3 55% of patients remain stable and 45% reported further reduction of the symptom.

**Figure 3:**
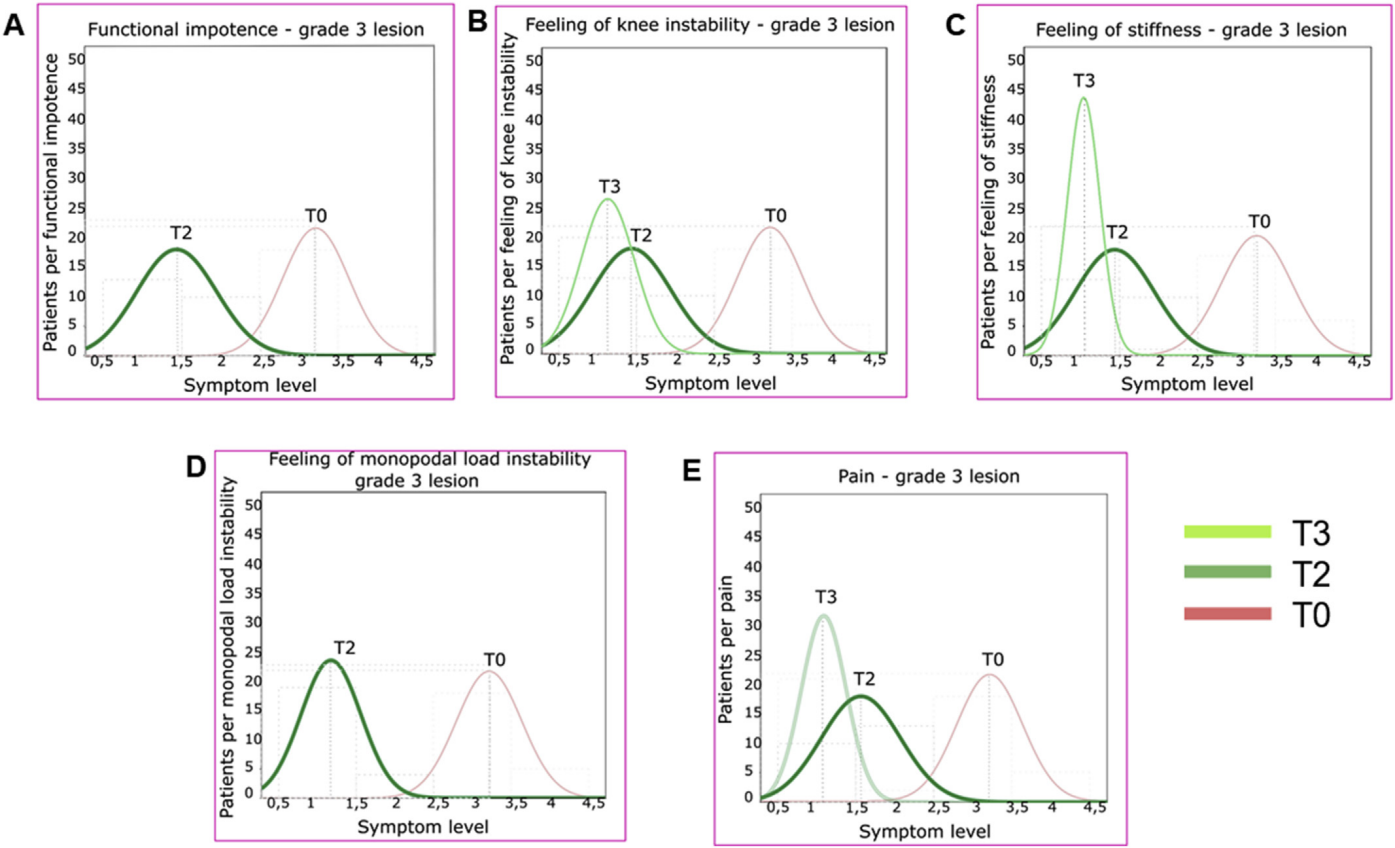
REAC efficacy in subjective assessments of symptoms represented by Gaussian distribution of 3 grade lesion patients from T0 to T3. REAC efficacy represented by the trend towards the distribution of patients on milder symptom levels.

**Figure 4:**
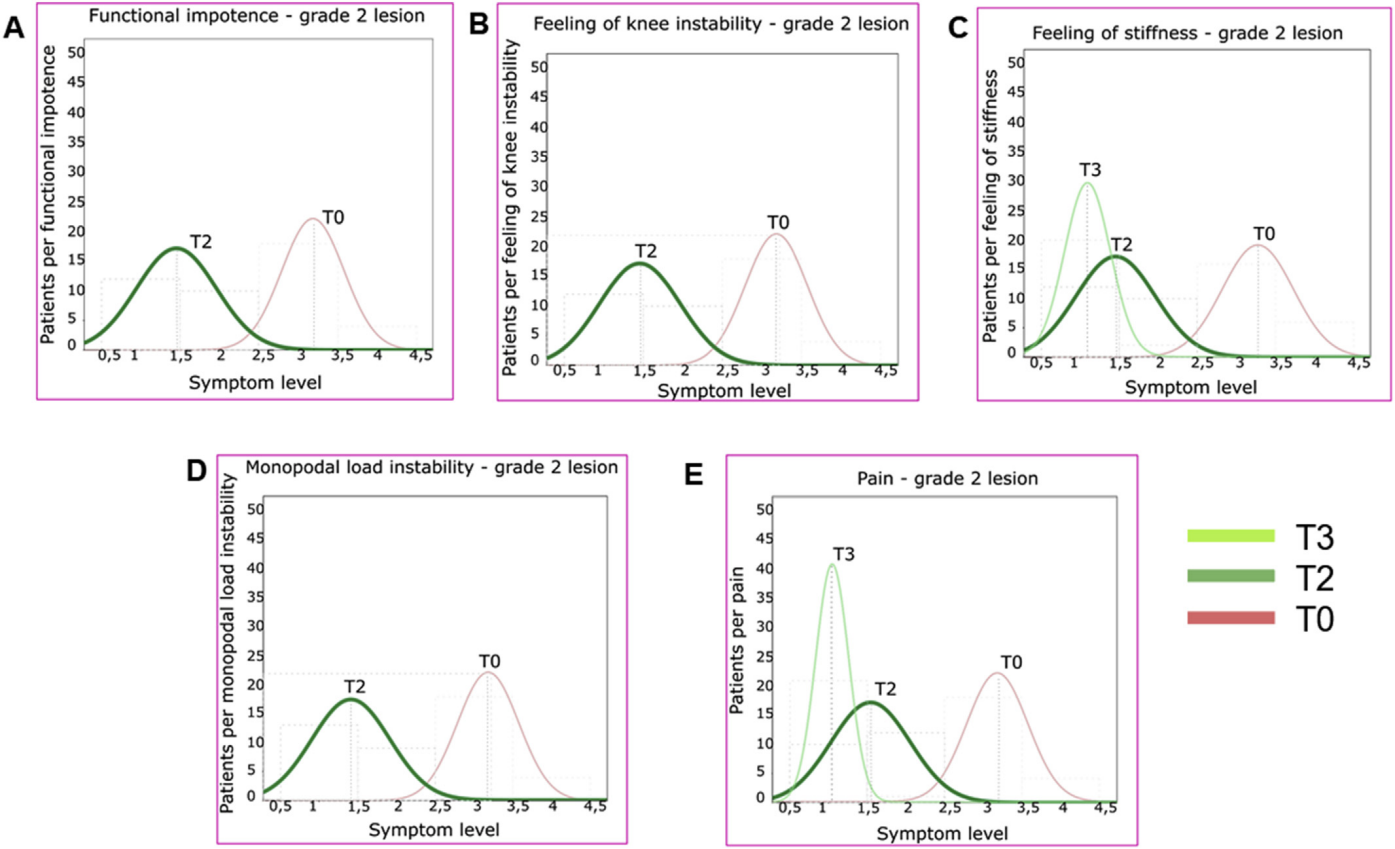
REAC efficacy in subjective assessments of symptoms represented by Gaussian distribution of grade 2 lesion patients from T0 to T3. REAC efficacy represented by the trend towards the distribution.

The efficacy of the REAC treatment is confirmed for both grades of lesion by the trend represented in the graphs of Figures 3A and 4A, where between T0 and T2 the Gauss curve moves towards milder symptom levels (abscissa values). At T3, the curve is not visible, since the totality of the patients showed full recovery and placed in correspondence of the absence of symptom in the values of the abscissa.

#### 3.2.2 Pain

At T0, the pain symptom was present in all the 45 patients.

At T2, self-assessment tests reported a reduction of the symptom in 100% of patients, while at T3 49% of patients (22 out of 43) reported further improvements.

Analysing the results by grade of lesion, in patients with grade 3 MCL lesion, at T2 REAC treatments have been shown to be effective in reducing the symptom pain in 100% of patients, while at T3 52% remain stable and 48% reported further reduction of the symptom.

As regards the patients with grade 2 MCL lesion, at T2 100% of patients reported a reduction of pain and at T3 50% remain stable and 50% reported further reduction of the symptom.

In the graphs of Figures 3E and 4E, for both grade 3 and 2 lesions, the Gauss Curves show a remarkable shift towards milder symptom levels (abscissa values) immediately after REAC treatment (T2), albeit with a non-homogeneous distribution of patients, and a further consolidation of the results at T3.

#### 3.2.3 Feeling of stiffness

At T0, the feeling of stiffness was present in all the 45 patients. At T2, REAC treatments have been shown to be effective in 100% of patients, while at T3 38% of patients (17 out of 43) reported further improvements.

In particular, at T2 100% of the patients with grade 3 MCL lesion reported a reduction of the symptom, while at T3 61% remain stable and 39% reported further reduction of the symptom.

As regards the patients with grade 2 MCL lesion, at T2, 100% of patients reported the reduction of feeling of stiffness and at T3 64% remain stable and 36% reported further reduction of the symptom.

In Figures 3C and 4C, for both grades of lesions the Gauss Curves show a remarkable shift towards milder symptom levels (abscissa values) immediately after REAC treatment (T2), and a further consolidation of the results at T3.

#### 3.2.4 Feeling of knee instability

At T0, all the 45 patients reported feeling of knee instability.

At T2, self-assessment tests reported a reduction of the symptom in 100% of patients, while at T3 38% of patients (17 out of 43) reported further improvements.

More in detail, in the group of patients with grade 3 MCL lesion, at T2 REAC treatments have been able to reduce the symptom in 100% of patients, while at T3 65% of patients remain stable and 35% reported further reduction of the symptom.

As regards the patients with grade 2 MCL lesion, at T2 100% of patients reported a reduction of feeling of knee instability, while at T3 55% remain stable and 45% reported further reduction of the symptom.

Figures 3B and 4B show that for both grades of lesion the Gauss curve moves remarkably towards milder symptom levels (abscissa values) immediately after the REAC treatment (T2) and the recovery of patients is confirmed at T3. In particular, as regards grade 2 MCL lesions, the T3 graph is not visible, since the totality of the patients showed full recovery and placed in correspondence of the absence of symptom in the values of the abscissa**.**

#### 3.2.5 Monopodal instability test

At T0, the subjective perception of instability, in monopodal instability test, was present in all the 45 patients.

At T2, REAC treatments have been shown to be effective in 100% of patients, while at T3 29% of patients (13 out of 43) reported further improvements.

Analysing the results by grade of lesion, in the group with grade 3 MCL lesion, at T2 about 100% of the patients reported a reduction of the symptom, while at T3 83% remain stable and 17% reported further reduction of the symptom.

As regards the patients with grade 2 MCL lesion, at T2 100% of patients reported the reduction of the subjective perception of monopodal instability, while at T3 59% remain stable and 41% reported further reduction of the symptom.

Figures 3D and 4D show that for both grades of lesion the Gauss curve moves remarkably towards milder symptom levels (abscissa values) immediately after the REAC treatment (T2) and the complete recovery of patients is confirmed at T3, as evidenced by the fact that the T3 graphs are not visible, since the totality of the patients placed in correspondence of the absence of symptom in the values of the abscissa.

## 4 Discussion

Traumatic events such as knee ligament injuries represent a stressful event affecting not only the joint stability, but also the psychological and physical condition of the subject. All that may affect the rehabilitation outcome, the complete functional recovery and performances.

The neuropsychical aspect that conditions post-traumatic recovery is increasingly described in the literature [9, 42]. In fact, although the rehabilitation techniques are much improved, the evidence of non-complete functional recovery after rehabilitation grows [43]. These findings are probably due to the fact that the unconscious neuropsychical altered component of the neuro psycho motor expression affects both the rehabilitation result and the emotional state of the patient, favouring psychological conditioning and attitudes of performance insecurity. This clinical study shows that the single administration of the REAC NPO neuromodulation treatment determines an immediate and long-lasting disappearance of Functional Dysmetria [18, 20], which represents one of the main signs of a neuro-psycho-physio-pathological and dysfunctional adaptive state [39]. This result demonstrates the efficacy of this neuromodulation treatment in inducing an initial improving of the neuropsychological status [24]. REAC NPO has been shown to have long-term effects [20] in the remodulation of the encephalic electro-metabolic activity, acting on the neurotransmission mechanisms and optimizing neuronal cell polarity processes, both in healthy subjects [18, 20] and in subjects also affected by severe neurodegenerative disorders [22, 23]. The results induced by the TO biomodulation treatment show its efficacy in facilitating, in the acute phase, the repair processes of damaged tissues. The physical treatments most widespread for this type of injury are used to promote reparative processes in damaged tissues, such as laser and diathermy treatments, which produce heat in tissues in order to facilitate reparative processes. The principle of action of REAC TO treatments is completely different, since they are not aimed at producing heat in the tissues, but at progressively recovering the endogenous bioelectric fields [44] at the cellular level, which are altered in the event of injury. In fact, when the integrity of a tissue is compromised by a lesion or trauma, there is an alteration of the cell polarity, which in turn alters the delicate mechanism of regulation of the electrochemical properties of the cells, inhibiting the production of ionic flows and therefore of the endogenous bioelectric fields underlying the reparative processes [44]. Therefore, REAC TO biomodulation treatments, through the progressive recovery of endogenous bioelectric fields, accelerate the reparative processes [44], avoid the chronicization of the inflammation, reduce oedema and pain, favouring the “restitutio ad integrum” of the injured tissue. The limits of this clinical study are the criteria of clinical evaluation that are subjective both by patients and evaluators, who in any case had extensive experience in this type of evaluation. As regards the absence of a control group, we can state that the scientific literature [1, 6] has well documented the course of these lesions with various types of treatments. In comparison with the results commonly found in clinical practice, the clinical course of all REAC NPO and TO treated patients was much faster, especially in functional recovery and in pain relief. At the end of the REAC treatments, completed within two weeks, all patients resumed the common activities, performed before the accident that had caused the MCL lesion, regardless of the grade of the lesion. Therefore, the comparison with the results obtained in the studies conducted with other treatments in the recovery of grade 3 and 2 MCL lesions, can represent a useful tool for checking the efficacy of the treatments proposed in this clinical study.

## 5 Conclusion

The results of this clinical study highlight the usefulness of therapeutic tools able to deal not only with the traumatic outcome, but also with the neuro psycho motor part of the trauma, that is an active part in influencing the recovery after trauma.

In fact, the complete recovery of the global function of the injured knee shows that REAC treatments can be effective on the physical response to trauma. Yet, the recovery has been in turn favoured by the remodulation of the altered neuropsychical component, which could determine insecurity and fear of re-injury, and induce dysfunctional motor strategies that have to be considered adaptive behaviours negatively conditioning the rehabilitation. The disappearance of the functional dysmetria, intended as disorder of the neuromotor control of the lower limbs, highlights that REAC treatment can act on the emotional altered component of the patient and on adaptive dysfunctional phenomena involving the execution of voluntary movements.

## Declarations

### Author contribution statement

A. Barcessat, M. Bittencourt, J. Pereira and A. Castagna: Conceived and designed the experiments; Performed the experiments; Analyzed and interpreted the data; Wrote the paper.

S. Rinaldi and V. Fontani: Conceived and designed the experiments; Analyzed and interpreted the data; Wrote the paper.

### Funding statement

This research did not receive any specific grant from funding agencies in the public, commercial, or not-for-profit sectors.

### Competing interest statement

The authors declare the following conflict of interests: S. Rinaldi and V. Fontani are the inventors of REAC technology.

### Additional information

No additional information is available for this paper.
